# London Hospital

**Published:** 1832-07

**Authors:** 


### HOSPITAL REPORTS.

### LONDON HOSPITAL.

### *Hydrophobia from the Bite of a Fox.**

EDWARD BOUTLE, aged forty-six, was admitted into the hospital, about nine o'clock on Sunday evening, June 10th, labouring under symptoms of hydrophobia. He was a man of intemperate habits, living in Wapping, and kept a favorite tame fox, with which he was accustomed to play. About six weeks ago the animal appeared unwell, and bit him slightly in the palm and back of his right hand. He took little notice of the bite, which got well in a short time: the animal, however, died two days afterwards, for which he was unable to account, but suspected that it had been poisoned. Saturday afternoon, June 9th, he complained of feeling dull and heavy, and of an aching pain in his fingers, which afterwards extended up the arm, and he felt an uneasiness just above the inner condyle and in the axilla. During the night he was restless and unable to sleep; the following morning (Sunday), in attempting to take a cup of tea, after swallowing about half, he threw it from him, and complained of its choking him. A constriction and uneasiness about the throat

* Medical Gazette.

came on; he was unwilling to swallow any thing; and being seen by a medical man, he was sent to the hospital in the evening.

When admitted, his look was wild, his respiration difficult, and he could not bear any one to approach him. He ran about the garden, and had a great horror of the warm air of the hospital. He was taken into one of the wards at a window, as he refused to pass through the passages; he was afraid of the slightest breath of wind, as when the door of the room was opened, or a person breathing upon him. His pulse was ninety-eight. Upon a glass of water being presented to him, he was thrown into a violent paroxysm. He complained of the uneasiness in his arm and pain in the shoulder. Ten grains of the carbonate of ammonia with one grain of opium, in pills, were proposed to be given to him every hour, but he dared not attempt to swallow them. An enema containing laudanum was ordered to be administered as soon as he should consent to it. A blister was applied in the course of the spine.

He passed a very distressing night; the paroxysms became more frequent and violent; he would lie down, and in about a minute start from the bed in painful convulsions, and continue walking about the room. He was perfectly sensible, and would talk; he said that he did not think that his present state had any thing to do with the bite, but he believed that he should not recover.

June 11th, nine A.M. He was now quite furious, rolling upon the floor and bed, threatening to destroy himself, and it was with difficulty that five men could restrain him. He was spitting his saliva violently from his mouth, and was in the greatest possible distress. He now consented to have an enema. An ounce of laudanum in some gruel was thrown up, and retained; in about an hour afterwards, an enema, with ʒij. of laudanum was given, and in half an hour was repeated. After the clyster he became more composed and quiet. He had afterwards two enemas, containing four drachms each.

Twelve o'clock. An ointment, consisting of two ounces of lard with two drachms of acetate of morphia, was rubbed upon the spine, but appeared rather to aggravate the spasms. He was now becoming weaker; the paroxysms were frequent, but less violent; he was delirious, and did not know his wife; pulse 160. From this time he gradually got worse, and died quite exhausted a little after two o'clock, about forty-three hours after the symptoms first appeared.

*Examination of the body twenty-four hours after death.* There was serous effusion between the arachnoid membrane and pia mater; the substance of the brain was increased in vascularity; there was also a considerable quantity of serum under the theca vertebralis. The superior cervical ganglion of the sympathetic, the eighth pair of nerves and phrenic nerves, were perfectly healthy in their appearance. The nerves were minutely traced to that part of the skin which was the seat of the bite, but nothing unusual was observed. The mucous membrane of the pharynx and larynx was remarkably vascular. The lungs were healthy, but rather gorged with blood.

